# Indications of Peptide Receptor Radionuclide Therapy (PRRT) in Gastroenteropancreatic and Pulmonary Neuroendocrine Tumors: An Updated Review

**DOI:** 10.3390/jcm10061267

**Published:** 2021-03-18

**Authors:** Baptiste Camus, Anne-Ségolène Cottereau, Lola-Jade Palmieri, Solène Dermine, Florence Tenenbaum, Catherine Brezault, Romain Coriat

**Affiliations:** 1 Gastroenterology and Digestive Oncology Unit, Cochin Hospital, AP-HP Centre, 27 rue du Faubourg Saint-Jacques, 75014 Paris, France; bcamus810@gmail.com (B.C.); lolajade.palmieri@aphp.fr (L.-J.P.); solene.dermine@aphp.fr (S.D.); catherine.brezault@aphp.fr (C.B.); 2 Faculté de Médecine, Université de Paris, 75006 Paris, France; annesegolene.cottereau@aphp.fr; 3 Department of Nuclear Medicine, Cochin Hospital, AP-HP Centre, 27 rue du Faubourg Saint-Jacques, 75014 Paris, France; florence.tenenbaum@aphp.fr

**Keywords:** neuroendocrine tumors, neoplasm metastasis, PRRT, peptide receptor radionuclide therapy, gastroenteropancreatic tumor, pulmonary tumor

## Abstract

Radionuclide therapy for neuroendocrine tumors is a form of systemic radiotherapy that allows the administration of targeted radionuclides into tumor cells that express a large quantity of somatostatin receptors. The two most commonly used radio-peptides for radionuclide therapy in neuroendocrine tumors are ^90^Y-DOTATOC and ^177^Lu-DOTATATE. Radio-peptides have been used for several years in the treatment of advanced neuroendocrine tumors. Recently, the randomized Phase III study NETTER-1 compared^177^Lu-DOTATATE versus high-dose (double-dose) octreotide LAR in patients with metastatic midgut neuroendocrine tumors, and demonstrated its efficacy in this setting. Strong signals in favor of efficiency seem to exist for other tumors, in particular for pancreatic and pulmonary neuroendocrine tumors. This focus on radionuclide therapy in gastroenteropancreatic and pulmonary neuroendocrine tumors addresses the treatment modalities, the validated and potential indications, and the safety of the therapy.

## 1. Introduction

Neuroendocrine tumors (NETs) are rare tumors characterized by the ability to synthesize, store, and secrete a variety of neuro-amines and peptides that can lead to secretory syndrome. NETs are mainly from the digestive tract and bronchopulmonary, and their incidence has been steadily increasing in the last three decades [1]. NETs are biological and clinically heterogeneous. The potential for metastatic evolution and the ability to generate a secretory syndrome vary considerably depending on the primary tumor location. For example, NETs of the small intestine have a higher malignant potential while appendix or gastric NETs malignant are potentials are close to zero. Metastatic NETs of the midgut often secrete serotonin and other vasoactive substances, resulting in a typical carcinoid syndrome, mainly characterized by hot flashes, diarrhea and right valvular heart disease. More than 40% of patients have metastatic disease at the time of diagnosis, which justifies the importance of a good pre-therapeutic evaluation. In recent years, randomized trials validated several new options such as targeted agents, including somatostatin analogues (SSA), everolimus and sunitinib. Since 1992, peptide receptor radionuclide therapy (PRRT) has been developed as a new therapeutic option in metastatic or non-resectable NET. This treatment corresponds to a form of systemic radiotherapy that allows targeted administration of systemic radiopharmaceuticals nucleides to tumor cells expressing high levels of somatostatin receptor (SSTR). For many years, evidence of an anti-tumor effect of PRRT were only obtained from non-randomized Phase II trials or retrospective studies. The NETTER-1 Phase III Randomized Trial finally validated this treatment option by confirming its low toxicity but also its effectiveness in tumor control. Following these results, ^177^Lu-DOTATATE was approved by the US Food and Drug Administration in 2018 and the European Medicines Agency in 2017 for the treatment of gastroenteropancreatic NETs that are well differentiated and obtain a prescription authorization in France in metastatic midgut NETs [2]. Some interesting data exist for pancreatic and pulmonary NETs. In this review, we discuss the clinical efficiency of PRRT in gastroenteropancreatic and pulmonary NETs.

## 2. Somatostatin Receptors (SSTR) and Radio-Labelled Somatostatin Analogues

Effect of PRRT is correlated with the ability for the markers to fix the SSTR (SSTR1-5). This therapy is therefore dedicated to NETs who strongly over expressed those receptors. SSTRs belong to a family of G-protein coupled receptors with seven transmembrane domains. The majority of well differentiated gastroenteropancreatic and pulmonary NETs are characterized by the strong expression of SSTR, including SSTR2 Grade 1/2 NETs express the SSTR more often and at higher levels than grade 3 NETs. When linked to the receptors, radiolabeled somatostatin analogues are internalized according to the normal recycling dynamics of membrane receptors and the degradation products of peptides are stored in lysosomes, in intracellular, which allows for the release and retention of radioactivity inside tumor cells [3]. This mechanism accounts for the low toxicity of the PRRT on the healthy cells. Radiolabelled somatostatin analogs are made up of an isotope radionuclide, a carrier molecule (derived from octreotide), and a chelating agent that binds them together and stabilizes the complex. The commonly used chelating agents are DOTA (DOTA acid and tetra-azacyclododecane-tetra-acetic) and DTPA (di-ethylenetriamine penta-aceticacid). Three radionucleides (^111^In, ^90^Y and ^177^Lu) were conjugated to the somatostatin analogues and their different physical characteristics confer specific advantages. The ^90^Y and ^177^Lu emit beta particles with higher energy and longer ranges, which translates into greater therapeutic potential. Due to the emission of gamma rays, the ^177^Lu can also be used for dosimetry and monitoring of tumor response [4].

## 3. Modalities of Realization and Patients Selection

**Figure 1:**
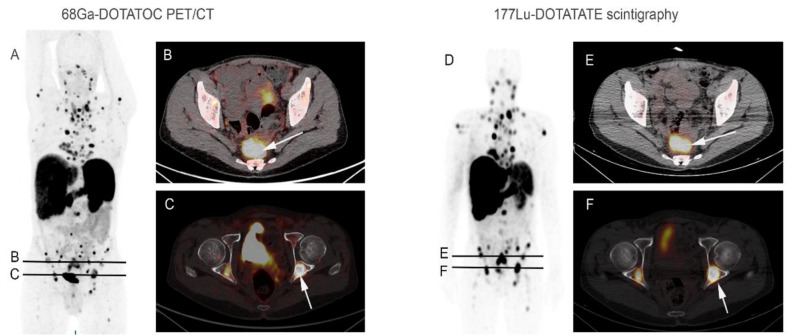
A 65-year-old man with metastatic well-differentiated grade 2 rectal neuroendocrine tumor, progressive after chemotherapy, addressed for PRRT. ^68^Ga-DOTATOC PET/CT maximum intensity projection image (**A**) and axial fused PET/CT images (**B**,**C**) showed high multiple focal uptakes, corresponding to mediastinal, abdominal lymph nodes, liver and bone lesions ((**C**) arrow: example of bilateral cotyle posterior wall lesions) associated with a large rectal lesion ((**B**) arrow). Post ^177^Lu-DOTATATE therapy whole-body image (**D**) after the first administration showed focal tracer uptake in all lesions correlating with ^68^Ga-DOTATOC PET/CT images, as observed in fused SPECT/CT images (**E**,**F**).

**Figure 2:**
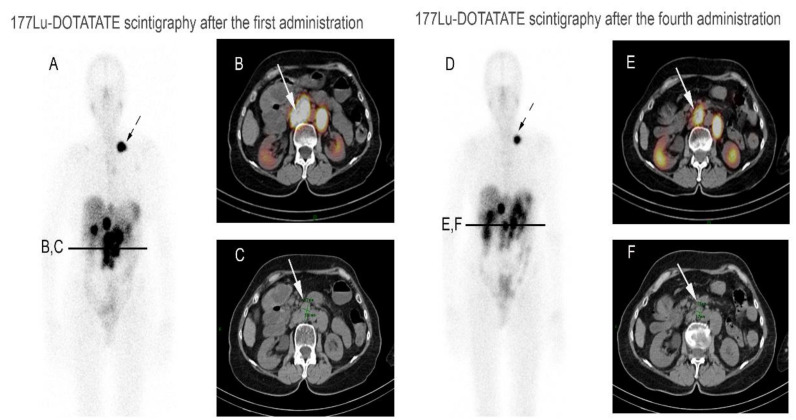
A 54-year-old woman with metastatic well-differentiated ileal neuroendocrine tumor, progressive under somatostatin analogs, addressed for PRRT. Post 177Lu-DOTATATE therapy whole-body image after the first administration (**A**) showed multiple foci of uptake including a large left subclavicular node (dotted arrow), liver lesions and large retroperitoneal lymph nodes (arrow) as illustrated in axial SPECT/CT fused (**B**) and CT (**C**) images. After the fourth administration, the treatment scintigraphy already detected partial response, with a decreased uptake in several lymph nodes (**D**), particularly in subclavicular (dotted arrow) and para-aortic sites (arrow), also associated with decreased in size, as demonstrated in axial SPECT/CT fusion (**E**) and CT (**F**) images.

PRRT is the single validated treatment option in the NETs, which for there is a predictive marker of answer: the expression of SSTR. Response rates have been shown to be increased in patients with a higher degree of absorption of radiotracers in the case of ^111^In-pentetreotid scintigraphy An overall response rate of approximately 60% has been reported in patients with a grade 4 according to the Krenning score, corresponding to a tumor absorption greater than that of the spleen, and kidneys. Intense fixation (SUV) greater than 16 on the ^68^Ga-DOTATOC PET/CT is a predictive marker of high tumor response (sensitivity: 95%; specficity: 60 %) [5] (Figure 1). The effectiveness of PRRT is correlated with the tumor volume and the location of the primary tumor (amount of SSTR present on the tumor cell) (Figure 2). Thus, NETs with high liver tumor volume are considered to be less sensitive to PRRT [6]. Similarly, it is suggested that pancreatic NETs frequently respond to the PRRT but with an earlier progression than in midgut NETs. The treatment modalities are relatively standardized in France with a treatment carried out by infusion of radiolabel split in four cycles spaced eight weeks apart. Administration concomitant positively charged aminoacids (lysine or arginine) is systematically carried out and reduces PRRT induced renal toxicity. The realization of PRRT requires a relatively normal renal function (glomerular filtration rate > 50 mL/min), a positive SSTR-based imaging (at least one grade 2 absorption according to Krenning’s score corresponding to equal or greater absorption to normal liver parenchyma) and normal medullary function.

## 4. Indications of PRRT in NETs

### 4.1. Gastrointestinal NET

Studies on the role of PRRT in Grade 1–2 gastrointestinal NETs were evaluated with two different plotters: ^90^Y-DOTATOC and ^177^Lu-DOTATATE. The use of ^90^Y-DOTATOC at a dose of 3.7 GBq/m^2^ in 1109 patients with gastrointestinal NET (*n* = 387), pancreatic (*n* = 342), pulmonary (*n* = 84) or other (*n* = 296) showed a morphologic response rate by 34% measured by a decrease in the sum of the longest diameters of all pretherapeutically detected tumor lesions. to the response assessment criteria usual radiological RECIST. In patients with intestinal and pancreatic NET, the levels of objective responses were 47% and 27%, respectively. In the overall population, the median of overall survival was 95 months [7]. The comparison clinical trials and retrospective series on the PRRT with the use of ^90^Y-DOTATOC in the gastroenteropancreatic NETs is impossible because the patient selection procedures, the criteria for fixation of the tracer to pre-therapeutic imaging, and infusion protocols differ widely in function of the studies. Currently, based on the NETTER-1 study, the ^177^LuDOTATATE- is the most widely used radiopeptide.

^177^Lu-DOTATATE is currently the more widely used radiopeptide. This radiolabel has demonstrated an efficiency similar to ^90^Y-DOTATOC, but with lower toxicity, especially hematologic (Table 1, [2,8,9,10,11,12,13,14]). In a serie of 310 patients treated for Gastroenteropancreatic NET per four cycles of 7.4 GBq of ^177^Lu-DOTATATE, it was found an objective response rate of 30%. Poor general condition (Karnofsky score < 70%) and liver damage were associated with an incorrect response to the treatment. For example, ^177^Lu-DOTATATE and ^90^Y-DOTATOC treatments are associated with poor results when tumor volume is high [15]. In a meta-analysis of 473 patients treated with ^177^Lu-DOTATATE for a NET, objective response rates ranged from 18% to 44% depending on the RECIST criteria, with an average disease control rate of 81% [16]. Retrospective and Phase II studies of ^177^Lu-DOTATATE showed a median progression-free survival of 33–36 months in patients with metastatic small size NET with documented tumor progression and/or an uncontrolled carcinoid symptom [8].

In this context, the NETTER-1 (Phase III randomized study) has evaluated and positioned the place of the PRRT at a very early stage in the management of patients with metastatical midgut NET [2]. This study compared 229 patients with advanced metastatic midgut NET, an Octreoscanner binding and progression under SSA at a fixed dose of 30 mg per month Octreotide, ^177^Lu-DOTATATE versus 60 mg per month of Octreotide (double dose). The study’s outcome was progression-free survival evaluated according to the radiological criteria RECIST 1.1. The treatment with ^177^Lu-DOTATATE resulted in a reduction of 79% of the risk of progression or death, compared to double-dose Octreotide (*p* < 0.0001). The median survival progression was not reached in the group treated with PRRT versus 8.4 months in the control group. Moreover, treatment with PRRT was associated with a rate of objective response of 18% versus 3% with Octreotide (*p* < 0.0004). Thus, and based on this Phase III data, ^177^Lu-DOTATATE is positioned as a new therapeutic option in grade 1–2 midgut NETs after disease progression under SSA and has been obtained marketing authorization for this indication. It is important to note that long-term follow-up is necessary in order to assess the impact of this new treatment on the overall patient survival. To date, the first data, obtained during the interim analyses suggested an overall survival benefit in the group treated by PRRT (*p* = 0.004).

More recently, some studies have investigated the role of PRRT in high-grade (G3, Ki-67 > 20%) gastroenteropancreatic (GEP) neuroendocrine neoplasms, called NEN G3. NEN G3 included well differentiated tumor (NET G3) and poorly differentiated (Neuroendocrine carcinoma, NEC), as described in the 2017 WHO classification for pancreatic NEN [17], with a similar expansion to gastrointestinal G3 tumors anticipated in the next WHO classification. In a study population of 19 well differentiated grade 3 NETs [9], a mean overall survival time of 38 months was observed. Other studies, including both NET G3, and NEC, have suggested a benefit on clinical outcome [18,19,20]. Particularly, in a multicenter retrospective study including 149 patients with GEP NEN G3, PRRT demonstrates promising response rates, disease control rates, PFS and OS [19] ^68^Ga-SSTR PET/CT and 18FDG PET/CT may also help to select the NET G3 patients who might benefit from PRRT. Indeed, a high SUV on SSTR PET/CT and no or minor 18F-FDG avidity appeared to be associated with a better prognosis [18].

### 4.2. Pancreatic NET

In pancreatic NETs, the effects are not confirmed by a controlled randomized Phase III study (Table 2). Available data consist of multiple single-arm prospective and retrospective trials. A Retrospective study series of 68 patients treated for pancreatic NET and having received four cycles of ^177^Lu-DOTATATE at 8 GBq showed no benefit in terms of survival in patients (*n* = 35) treated with PRRT on the front line [21]. On the other hand, signals in favor of an efficiency are identified. In this cohort a disease control rate of 85% and a median time to progression of 34 months was observed [6].

Other retrospective studies argue for efficacy in disease control and low toxicity of PRRT in pancreatic NETs. A retrospective series of 443 gastroenteropancreatic and pulmonary neuroendocrine tumours, including 133 pancreatic NETs, showed a disease control rate of 84% and an objective response rate of 54% in the pancreatic tumour subgroup. The median progression-free survival was 30 months and overall survival was 71 months in this subgroup. Short-term tolerability was marked by nausea, vomiting, and abdominal pain (related to the prior infusion of amino acids during the ^177^Lu DOTATATE treatment to decrease absorption, and thus toxicity, in the kidney). No short-term hematotoxicity or renal toxicity was found in this series. Long-term toxicity was marked by four cases of acute leukemia (0.7% of the study population) and nine cases of myelodysplastic syndrome (1.5%). [10].

A meta-analysis comparing the efficacy of PRRT with ^177^Lu DOTATATE and everolimus (one of the therapeutic alternatives to PRRT in gastroenteropancreatic NET not accessible to surgical treatment) compared 15 articles reporting 697 patients treated with PRRT and 12 articles reporting 946 patients treated with everolimus. The objective response rate was higher with PRRT compared to everolimus (47% vs. 12% respectively, *p* < 0.001), as was the rate of disease control (81% vs. 73% respectively, *p* < 0.001), and progression-free survival (25.7 months vs. 14.7 months respectively, *p* < 0.001). The tolerance profile was also better with PRRT than everolimus. Grade 3/4 hematotoxicity was found in 5% of cases with PRRT versus 11% with everolimus (*p* = 0.02). This result is less contrasted for grade 3/4 nephrotoxicity found in 1% of PRRT and 2.5% of everolimus treatments (*p* = 0.34). The number of treatment interruptions related to its toxicity was 59 in the everolimus arm versus 0 in the PRRT arm [22]. These findings suggest that PRRT is effective in controlling disease in patients who are not amenable to surgical treatment, as well as less toxicity (Table 2, [6,9,10,11,12,13,21,23,24,25]) compared to other treatment alternatives. Nevertheless, these data for pancreatic NETs need to be confirmed by a prospective, randomized, double-blind, phase 3 study, similar to the NETTER-1 trial for NET in the small intestine. A possible role for PRRT as a neoadjuvant agent has been suggested by case reports or small case series, but need to be further explored [26]

Furthermore, ^90^ Y-DOTATOC have also been tested prospectively in panNET [27] mainly by a phase 2 trial including 342 patients with an ORR of 47% [7]. Pancreatic NETs are more sensitive to the cytotoxic chemotherapy than other NETs. Thus, the place of the PRRT in the treatment strategy has to be evaluated.

In summary, the PRRT is perfectly positioned in midgut NETs progressing under SSA and has yet to find its place in pancreatic NETs.

**Table 2:** Studies reporting 177 Lu-DOTATATE PRRT efficacy and tolerance in pancreatic NETs.

Type of Study	Reference	Total Population	panNET Subgroup	Response Criteria	CR *n*(%)	PR *n*(%)	MR *n*(%)	SD *n*(%)	PD *n*(%)	ORR *n*(%)	DCR *n*(%)	PFS	OS	Grade 3–4 Toxicity *n*(%)
												Median in Months (95% CI)		
Phase 1/2	Bodei 2011 [11]	unresectable or metastatic tumor (*n* = 51)	*n* = 14	RECIST modified *	0(0)	8(57)	1(7)	2(14)	3(21)	9(64)	11(79)	NS	NS	HematoT: 2 (4)
Phase 2	Sansovini 2013 [23]	advanced G1/G2 panNET (*n* = 52)	*n* = 52	SWOG	4(8)	11(21)	N/A	27(52)	10(19)	15(29)	42(81)	29 (19–39)	NR	0%
Retrospective	Ezzedin 2014 [21]	metastatic G1/G2 panNET (*n* = 68)	*n* = 68	RECIST v1.1	0(0)	39(57)	N/A	19(28)	10(15)	39(57)	58(85)	NS	NS	HematoT: 6% NephroT: 0%
				SWOG modified *	0(0)	41(60)	8(12)	9(13)	10(15)	49(72)	58(85)	34 (26–42)	53 (46–60)	
			baseline SD (*n* = 22)		NS	NS	NS	NS	NS	NS	19(86)	NS	48 (43–52)	
			baseline PD (*n* = 46)		NS	NS	NS	NS	NS	NS	39(85)	NS	54 (46–61)	
			Non functional (*n* = 50)		NS	NS	NS	NS	NS	NS	44(88)	NS	63 (48–78)	
			Functional (*n* = 18)		NS	NS	NS	NS	NS	NS	14(78)	NS	45 (37–53)	
Retrospective	Ezziddin 2014 [6]	G1/G2 GEP NET (*n* = 74)	*n* = 33	SWOG modified *	0(0)	18(55)	6(18)	6(18)	3(9)	24(77)	30(91)	25(17–33)	57 (48–66)	NS
Retrospective	Brabander 2017 [10]	GEP and bronchial NET (*n* = 443)	*n* = 133	RECIST v1.1	6(5)	66(50)	N/A	40(30)	17(13)	72(54)	112 (84)	30	71	AL: 4 (0.7) MDS: 9 (1.5) NephroT: 0
			-baseline SD (*n* = 21)		1(5)	9(43)	N/A	10(48)	1(5)	10(48)	20(95)	31	NR	
			-baseline PD (*n* = 66)		2(3)	36(55)	N/A	15(23)	10(15)	38(58)	53(80)	31	71	
			Functional (*n* = 21)		1(5)	12(57)	N/A	4(19)	3(14)	13(62)	17(81)	30	NR	
			Non functional (*n* = 112)		5(4)	54(48)	N/A	36(32)	14(13)	59(53)	95(85)	30	69	
Expanded access trial	Hamiditabar 2017 [12]	NET with baseline progressive disease (*n* = 144)	*n* = 48	RECIST	0(0)	6(13)	N/A	18(38)	23(48)	6(13)	24(50)	NS	NS	HematoT: 16 (11) HepatoT: 3 (3) NephroT: 0
Phase 2	Sansovini 2017 [24]	unresectable or metastatic G1/G2 panNET baseline PD (*n* = 60)	*n* = 60	SWOG	4(7)	14(23)	N/A	31(52)	11(18)	18(30)	49(82)	29 (20–54)	NR	HematoT: 0 NephroT: 1.6%
Prospective	Garske-Roman 2018 [13]	metastatic NET (*n* = 200)	panNET or Duodenal NET (*n* = 49)	RECIST v1.1	1(2)	21(43)	N/A	24(49)	2(4)	22(45)	46(94)	27 (17–33)	42 (31–NR)	AL: 3(1.5) HematoT: 30(15) NephroT: 1(0.5)
			Functional panNET or Duodenal NET(*n* = 20)		1(5)	8(40)	N/A	11(55)	0(0)	9(45)	20 (100)	24 (12–37)	39 (24–53)	
			Non functional panNET or Duodenal NET(*n* = 29)		0(0)	13(45)	N/A	13(45)	2(7)	13(45)	26(90)	27 (14–33)	NR	
Retrospective	Demirci 2018 [9]	Unresectable or metastatic G1–G3 NET (*n* = 186)	*n* = 62	RECIST	3(5)	35(56)	N/A	5(8)	19(31)	38(61)	43(69)	Mean 42 (35–49)	Mean 57 (52–62)	HematoT: 2(1) NephroT: 2(1)
Retrospective	Zandee 2019 [25]	Metastatic functional G1/G2 panNET (*n* = 34)	*n* = 34	RECIST	1(3)	19(56)	N/A	8(24)	6(18)	20(59)	28(82)	18 (3–36)	NR	HematoT: 15% MDS (3%)
Meta-analysis	Satapathy 2019 [22]	Advanced G1–G3 panNET (*n* = 674)	*n* = 674	RECIST SWOG WHO	NS	NS	NS	NS	NS	47%	546 (81)	26 (19–32)	NR	HematoT 5% (0.3–15%) NephroT 1%

PRRT: Peptide receptor radionuclide therapy. NET: Neuroendocrine tumor. GEP: Gastroenteropancreatic. CI: Confidence interval. CR: Complete response. PR: Partial response. MR: Minor response. SD: Stable disease. PD: Progressive disease. ORR: Objective response rate. DCR: Disease controle rate (defined as the sum of complete, partial, minor responses and stable disease), PFS: Progression free survival, OS: Overall survival, N/A: Not applicable, NS: Not stated, NR Not reached, HematoT:hematotoxicity, NephroT: nephrotoxicity, AL: acute leukemia, MDS: myelodysplastic syndrome. Months and percentages reported to zero decimal places. * include Minor response. All responses indicated are for the subgroup of pancreatic NETs.

### 4.3. Pulmonary NET

Lung NET are well differentiated neuroendocrine tumors (NET) classified as typical carcinoids (TC: Ki-67 of up to 5%) or atypical carcinoids (AC: Ki-67 of up to 20%). Several studies have evaluated PRRT in patients with pulmonary NETs and have reported similar results to those observed in midgut NETs (Table 3, [9,10,11,12,13,28,29]). However, the indications are limited because several pulmonary NETs, especially carcinoids atypical tumors (AC), express relatively few SSTR2 and are therefore ineligible for this form of treatment [30]. In a study of 34 patients with TC and AC (56%) treatment with ^177^Lu-DOTATATE was associated with a low objective response rate of 15%. The median progression-free survival and overall survival were 19 and 49 months, respectively [28]. 

Limited but promising data exists on ^90^Y-DOTATOC efficacy from 3 trials which have included 12 bronchial NEN patients, with a 100% DCR reported using WHO tumor response criteria, with the ORR ranging from 0% to 50% [31].

Recently, radio tracer activity (^177^Lu-DOTATATE and ^90^Y-DOTATOC) has been evaluated in single center series of 114 patients with pulmonary NET. The median progression-free- and overall survivals were 28 and 59 months, respectively. Nephrotoxicity was more frequently observed in patients who received ^90^Y-DOTATOC [29]. Thus, to date, we have a bundle of arguments in favor of an effective of PRRT in the pulmonary NETs. These data argue for a phase III study in this situation.

### 4.4. Retreatment PRRT after PRRT Failure

All patients treated with PRRT will experienced a progression of the disease within a few months to a few years. In the NETTER-1 study, only 30% of the patients had a progression of their disease at 20 months. The toxicity and efficacy of retreatment with additional cycles of PRRT have been evaluated in several small retrospective series. Twenty-seven patients progressing after an initial response to ^90^Y-DOTATOC have been treated again with^177^Lu-DOTATATE [32]. The rate of disease control was 70% and no serious toxicity has been reported [32]. More recently, a phase II study focused on retreatment with^177^Lu-DOTATATE at low doses (up to 18.5 GBq administered in 4–5 cycles) in 26 patients who progressed at least 12 months after starting treatment with ^90^Y-DOTATOC. The median progression-free survival was 22 months, and the disease control rate was 85% [33]. Overall, PRRT retreatment in NETs appeared to be associated with low toxicity, a lower tumor burden and a lower progression-free survival than the first PRRT treatment. The retreatment may be discussed again in patients with a very good tolerance of the initial treatment and a prolonged response. Future studies will be required in order to identify the place for PRRT retreatment in comparison to validated treatments (targeted therapies, chemotherapy).

### 4.5. Side Effects of PRRT

PRRT is generally well tolerated, particularly with ^177^Lu, which appeared safer than Y90 both in terms of haematological/renal toxicity and outcomes. The better tolerance of 177Lu explains why it has been preferred in many studies.

#### 4.5.1. Subacute Effects

PRRT is overall well tolerated with most patients experiencing only moderate toxicity. In the study NETTER-1, validating the PRRT in midgut NETs, 5% of the patients discontinued treatment due to toxicity related to PRRT. Acute side effects are mainly nausea, vomiting, fatigue and abdominal pain. These adverse events are mainly caused by the simultaneous infusion of aminoacids. Particular vigilance must be exercised with regard to the risk of carcinoid crisis. This event was reported in a very small minority (1%) of patients who received an PRRT treatment. This crisis usually appears within 48 h of the first infusion and is related to the massive release of active amines [34]. A myelosuppressure has been described and is caused by the irradiation of the bone marrow. Bone marrow is particularly radiosensitive, and this toxicity does not appear to be associated to the expression of SSTRs in myeloid cells. Myelosuppression generally develops four at six weeks after the infusion, is usually grade 1/2 and is reversible. Hematotoxic effects grade 3 and 4 have been described in 13% and 10% of the patients receiving ^90^Y-DOTATOC and ^177^Lu-DOTATATE, respectively [35]. Lymphopenia is the more often reported severe cytopenia. Some studies have also reported that PRRT could be safely used in the case of diffuse metastatic bone marrow involvement, without irreversible myelosuppression, although a higher incidence of subacute transient hematologic toxicity was observed [36]

Other toxicities have been reported, including a risk of hepatotoxicity in the patients with major liver involvement. Thus, as indicated above, it is desirable to consider PRRT early in the management process of the disease.

#### 4.5.2. Long-Term Effects

Long-term side effects of PRRT can include renal failure and leukemic or myelodysplastic syndromes.

The radiolabels are reabsorbed in the proximal tubules, may accumulate in the proximal tubules in the renal interstitium and cause kidney damage. Because of its higher energy and longer penetration range, ^90^Y irradiates the renal interstitium glomeruli more extensively than ^177^Lu. In a large institutional series of 1109 patients treated with ^90^Y-DOTATOC, 103 patients (9%) were treated with ^90^Y-DOTATOC with severe renal toxicity [7]. Nowadays, the concomitant administration positively charged aminoacids results in a reduction of up to 40% of the renal absorption. Despite renal protection, the median decrease of the creatinine clearance is estimated to 4% per year in patients treated with ^177^Lu-DOTATATE. The risk factors for nephrotoxicity cited are diabetes and poorly controlled hypertension. Risk of severe nephrotoxicity (grade 3/4) was observed in only 1.5% of patients [37]. In overall, end-stage renal disease as a consequence of PRRT is extremely rare.

Cases of leukemia and myelodysplastic syndromes have been reported as late-onset PRRT toxicity, with an estimated incidence of up to 2% [37,38]. Age over 70 years, cytopenia before treatment, the presence of bones metastasis, the high number of previous treatments, the prior use of an alkylating agent and the radiotherapy increases the risk of secondary myelodysplastic syndrome. Brieau et al. reported in a retrospective monocentric study conducted in a population of patients treated with PRRT and pre-treatment alkylating chemotherapy an increased risk late hematologic toxicity (20%; four patients out of 20) [39]. The main alkylating agent used in the NET treatment is temozolomide. The development of myelodysplastic syndrome or leukemia has been reported between 30 and 70 months after treatment with PRRT [39]. These results suggest an imputability of alkylating agents associated with PRRT as only 1% of the patients treated only by alkylating chemotherapy developed myelodysplastic syndrome. This difference in rates reported by Brieau et al. and the different retrospective studies could be linked to the performing primary chemotherapy. These data are in favor of PRRT treatment prior to chemotherapy.

Whereas a blood RNA assay has been developed to predict tumour response to PRRT, no molecular markers have been found to predict PRRT toxicity [40].

## 5. Perspectives, Ongoing Studies

The role of PRRT in NETs is evolving. Randomized controlled trials are ongoing and will probably consolidate PRRT: in GEP-NETs versus other standard of care treatment such as Everolimus (Compete trial), in Pan-NET versus Sunitinib [41] and in G2–G3 NETs as upfront treatment versus Octreotide LAR [42].

Some new strategies are also under investigation: combination with chemotherapy (ex: with Capecitabine for aggressive FDG-positive G1–G3 GEP-NETs), using new theranostics agents (SS-Antagonist) and alpha-PRRT [43]. Intra-arterial administration has been also proposed and could be combined with intravenous administrations, as tested in a phase 1 trial for patients with liver-dominant metastatic pancreatic NETs by Bodei and colleagues [44].

## 6. Conclusions

PRRT has anti-tumor efficacy in NETs with a benefit in terms of objective response rate and survival without progression. The level of evidence varies depending on the location of the primary lesion. The NETTER-1 trial, a randomized Phase III study, validated the place of PRRT early in midgut NETs. In other localizations, specifically lung and pancreas, although data from controlled randomized trials are lacking, several studies argue for the effectiveness of the PRRT legitimating PRRT as a possible option in patients with SSTR-positive tumors [42,45]. Prospective studies are needed to establish the appropriate timing in the treatment algorithm vs the others validated therapeutics in NET (chemotherapy, everolimus and sunitinib for pancreatic NENs), depending on tumor localization. A key challenge remains to identify biomarkers, from imaging and molecular data, to predict PRRT response, towards a personalized treatment plan.

## Figures and Tables

**Table 1:** Studies reporting ^177^Lu-DOTATATE PRRT efficacy and tolerance in midgut NETs.

Type of Study	Reference	Total Population	Midgut NET Subgroup	Response Criteria	CR *n*(%)	PR *n* (%)	MR *n*(%)	SD *n*(%)	PD *n*(%)	ORR *n*(%)	DCR *n*(%)	PFS	OS	Grade 3–4 Toxicity *n*(%)
												Median in Months (95% CI)		
Phase 1/2	Bodei 2011 [11]	unresectable or metastatic tumor (*n* = 51)	*n* = 19	RECIST modified *	0(0)	2(10)	6(32)	7(37)	4(21)	2(11)	15(80)	NS	NS	HematoT: 2(4)
Retrospective	Sabet 2015 [8]	Unresectable, metastatic G1/G2 midgut NET (*n* = 61)	*n* = 61	SWOG modified * RECIST v1.1	0(0)	8 (13)	19 (31)	29(48)	5(8)	8(13)	56(92)	33(25–41)	61 (N/A)	HematoT: 5(8) NephroT: 0(0)
Phase 3	Strosberg 2017 [2]	Unresectable or metastatic G1/G2 midgut NET progressive under octreotide LAR (*n* = 229)	*n* = 201 evaluable for objective response	RECIST v1.1	1(1)	17 (17)	N/A	60(60)	23(23)	18(18)	78(78)	65(50–77) **	NS	HematoT: 11(5) NephroT: 0(0)
Expanded access trial	Hamiditabar 2017 [12]	NET with baseline progressive disease (*n* = 144)	*n* = 53	RECIST	0(0)	2 (4)	N/A	32(60)	19(36)	2(4)	34(64)	NS	NS	HematoT: 16 (11) HepatoT: 3(3) NephroT: 0(0)
Retrospective	Brabander 2017 [10]	GEP and bronchial NET (*n* = 443)	*n* = 181	RECIST v1.1	2(1)	55 (30)	N/A	99(55)	16(9)	57(31)	156(86)	30	60	AL: 4(0.7) MDS: 9(1.5) NephroT: 0(0)
			with baseline SD (*n* = 32)		0(0)	10 (31)	N/A	18(56)	3(9)	10(31)	28(87)	24	82	
			with baseline PD (*n* = 94)		1(1)	28 (30)	N/A	50(53)	9(10)	29(31)	79(84)	29	50	
Retrospective	Yalchin 2017 [14]	metastatic midgut NET (*n* = 133) ***	*n* = 133	RECIST v1.1	0(0)	12 (9)	N/A	67(50)	54(41)	12(9)	79(59)	29	34	NS
Prospective	Garske Roman 2018 [13]	metastatic NET (*n* = 200)	*n* = 108	RECIST v1.1	0(0)	13 (12)	N/A	87(79)	2(2)	13(12)	100(91)	29(23–35)	48(40–60)	AL: 3(1.5) HematoT: 30(15) NephroT:1(0.5)
Retrospective	Demirci 2018 [9]	Unresectable or metastatic G1–G3 NET (*n* = 186)	*n* = 42	RECIST	2(5)	17 (41)	N/A	15(37)	7(17)	19(46)	34(83)	38(31-44)	57(54-61)	HematoT: 2(1) NephroT: 2(1)

PRRT: Peptide receptor radionuclide therapy. NET: Neuroendocrine tumor. GEP: Gastroenteropancreatic. CI: Confidence interval. CR: Complete response. PR: Partial response. MR: Minor response. SD: Stable disease. PD: Progressive disease. ORR: Objective response rate. DCR: Disease controle rate (defined as the sum of complete, partial, minor responses and stable disease), PFS: Progression free survival, OS: Overall survival, N/A: Not applicable, NS: Not stated, NR Not reached, LAR: Long acting repeatable. HematoT:hematotoxicity, NephroT: nephrotoxicity, AL:acute leukemia, MDS: myelodysplastic syndrome. Months and percentages reported to zero decimal places. * include Minor response ** PFS estimated. Median PFS not reached at the time of the analysis. *** Mixed 90Y and 177Lu DOTATATE, 83 and 17% respectively.

**Table 3:** Studies reporting 177 Lu-DOTATATE PRRT efficacy and tolerance in bronchopulmonary NETs.

Type of Study	Reference	Total Population	Bronchial NET Subgroup	Response Criteria	CR *n*(%)	PR *n*(%)	MR *n*(%)	SD *n*(%)	PD *n*(%)	ORR *n*(%)	DCR *n*(%)	PFS	OS	Grade 3–4 Toxicity *n*(%)
												Median in Months (95% CI)		
Phase 1/2	Bodei 2011 [11]	unresectable or metastatic tumor (*n* = 51)	*n* = 5	RECIST modified *	0(0)	2(40)	2(40)	1(20)	0(0)	2(40)	5(100)	NS	NS	HematoT: 2(4)
Phase 2	Ianniello 2016 [28]	unresectable or metastatic bronchial carcinoids NET (*n* = 34)	*n* = 34	SWOG	1(3)	4(12)	N/A	16(47)	13(38)	5(15)	21(62)	19 (13–26)	49 (26–69)	HematoT: 0(0) NephroT: 0(0)
Retrospective	Mariniello 2016 [29]	unresectable or metastatic bronchopulminar carcinoid NET (*n* = 114)	*n* = 114	RECIST modified *	0(0)	15(13)	15(13)	46(41)	38(33)	30(26)	76(67)	28 (15–45)	59 (32–92)	HematoT: 7(6) NephroT: 0(0)
Expanded access trial	Hamiditabar 2017 [12]	baseline progressive disease (*n* = 144)	*n* = 14	RECIST	0(0)	2(14)	N/A	6(43)	5(36)	2(14)	8(60)	NS	NS	HematoT: 16(13) HepatoT: 3(3) NephroT: 0(0)
Retrospective	Brabander 2017 [10]	GEP and bronchial NET (*n* = 443)	*n* = 23	RECIST v1.1	0(0)	7(30)	N/A	7(30)	6(26)	7(30)	14(61)	20	52	AL: 4(0.7) MDS: 9(2) NephroT: 0(0)
Prospective	Garske Roman 2018 [13]	Metastatic NET (*n* = 200)	*n* = 6	RECIST v1.1	0(0)	1(17)	N/A	5(83)	0(0)	1(17)	6(100)	18 (12–43)	NR (19–NR)	AL: 3(1.5) HematoT: 30(15) NephroT:1(0.5)
Retrospective	Demirci 2018 [9]	Unresectable or metastatic G1-G3 NET (*n* = 186)	*n* = 22	RECIST	0(0)	9(41)	N/A	4(18)	9(41)	9(41)	13(59)	32 (24–40)	44 (37–52)	HematoT: 2(1) NephroT: 2(1)

PRRT: Peptide receptor radionuclide therapy. NET: Neuroendocrine tumor. GEP: Gastroenteropancreatic. CI: Confidence interval. CR: Complete response. PR: Partial response. MR: Minor response. SD: Stable disease. PD: Progressive disease. ORR: Objective response rate. DCR: Disease controle rate (defined as the sum of complete, partial, minor responses and stable disease), PFS: Progression free survival, OS: Overall survival, N/A: Not applicable, NS: Not stated, NR Not reached, HematoT:hematotoxicity, NephroT: nephrotoxicity, AL: acute leukemia, MDS: myelodysplastic syndrome. Months and percentages reported to zero decimal places. * include Minor response All responses indicated are for the subgroup of bronchopulmonary NETs.

## Abbreviations

PRRT	Peptide Receptor Radionuclide Therapy
SSTR	Somatostatin Receptor
RECIST	Response evaluation in solid tumors
SUV	Standardize Uptake Value
LAR	Long acting release
NET	Neuroendocrine tumor
SSA	Somatostatin Analogs
GBq	Giga Becquerel
